# Duodenal Villous Atrophy in a TTG-Negative Patient Taking Olmesartan: A Case Report and Review of the Literature

**DOI:** 10.1155/2016/6091571

**Published:** 2016-04-14

**Authors:** Tasha Kulai, Thomas Arnason, Donald MacIntosh, John Igoe

**Affiliations:** ^1^Department of Medicine, Dalhousie University, Halifax, NS, Canada B3H 2Y9; ^2^Division of Anatomical Pathology, Dalhousie University, Halifax, NS, Canada B3H 2Y9; ^3^Division of Digestive Care and Endoscopy, Dalhousie University, Halifax, NS, Canada B3H 2Y9

## Abstract

Olmesartan, an angiotensin II receptor antagonist used to treat hypertension, is associated with few adverse effects. Here, a case of severe sprue-like enteropathy and acute kidney injury is described in a 68-year-old male taking olmesartan for 3-4 years. He presented to hospital with a five-week history of diarrhea, vomiting, and a 20 lb weight loss. Anti-TTG was negative with a normal IgA. Biopsies of the distal duodenum and duodenal cap revealed marked blunting of the villi with near complete villous atrophy of the biopsies from the bulb. There was an increase in intraepithelial lymphocytes as well as neutrophils in the surface epithelium. The patient's diarrhea improved upon discontinuation of olmesartan and he returned to his previous weight. Repeat endoscopy four months later demonstrated complete resolution of inflammatory change with normal villous architecture. Long-term olmesartan use is associated with severe sprue-like enteropathy. The mechanism of intestinal injury is unknown. Duodenal biopsy results may mimic other enteropathies such as celiac disease. Physicians should consider medications as potential etiologies of enteropathy.

## 1. Case Presentation

**Figure 1:**
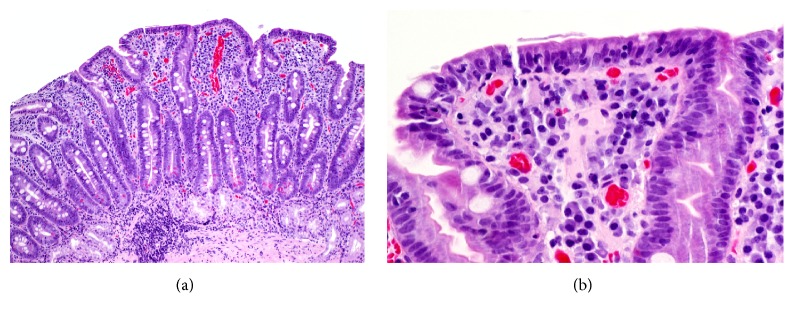
(a) Biopsy of the duodenum reveals villous blunting and expansion of the lamina propria inflammatory infiltrate (haematoxylin and eosin, original magnification ×100). (b) Higher power view of the surface epithelium from (a) shows increased intraepithelial lymphocytes (haematoxylin and eosin, original magnification ×400).

A 68-year-old male presented to his family physician with a five-week history of nonbloody diarrhea, vomiting, and a 20 lb weight loss. Three days previously he had been seen by ophthalmology with new onset right eye pain, redness, and light sensitivity and diagnosed with severe right nongranulomatous anterior uveitis. He was treated with homatropine, dexamethasone, and prednisolone eye drops. He had no fevers, joint pain, skin changes, or recent travel. Past medical history included kidney stones, hypertension, and bioprosthetic aortic valve replacement three years earlier for severe aortic stenosis. He had been on olmesartan/hydrochlorothiazide 40/12.5 mg daily for 3-4 years. Other medications included ASA 81 mg twice weekly, vitamin C daily, multivitamin daily, cod liver oil daily, and acetaminophen as needed. Blood work ordered by his family physician included an elevated creatinine at 474 *μ*mol/L. He was sent to the emergency department and admitted for an acute kidney injury presumably secondary to dehydration. Physical examination was unremarkable. He had normocytic anemia (hemoglobin 120 g/L), normal albumin (40 g/L), normal electrolytes, and nonanion gap metabolic acidosis. Creatinine improved to 77 *μ*mol/L with intravenous fluids over 5 days. Stool was negative for culture, parasites, and* Clostridium difficile*. Anti-tissue transglutaminase (TTG) antibody was negative with normal immunoglobulin A levels. Biopsies of the distal duodenum and duodenal cap revealed marked villous blunting with near complete villous atrophy of the small intestinal mucosa in some areas (Figure 1). There was an increase in intraepithelial lymphocytes as well as neutrophils in the surface epithelium. The crypts had a prominent increase in apoptosis.

In hospital, his uveitis improved considerably and he was reassessed by ophthalmology. A diagnosis of idiopathic bilateral anterior and intermediate uveitis was confirmed after negative workup for syphilis, Lyme disease, sarcoid, and tuberculosis. The uveitis resolved at 16 weeks with a taper of prednisolone eye drops.

**Figure 2:**
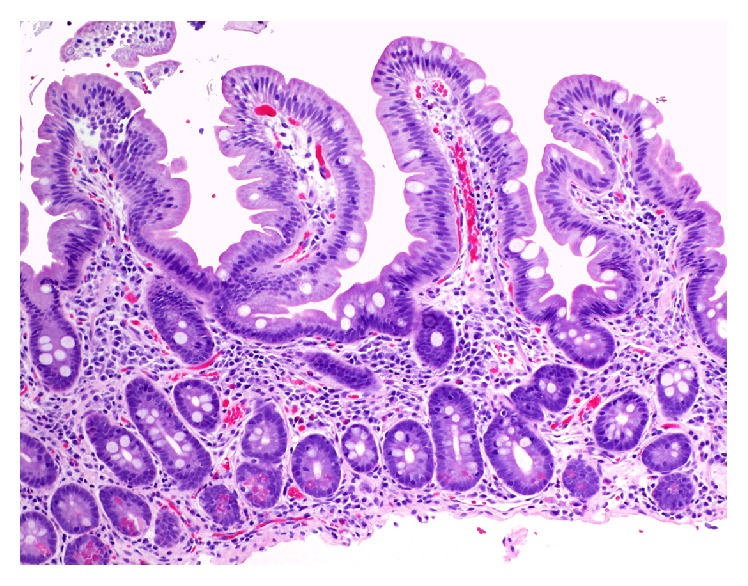
Follow-up biopsy 14 weeks after discontinuation of olmesartan shows striking normalization of the duodenal histology. There is normal villous architecture and no increase in intraepithelial lymphocytes in the biopsy (haematoxylin and eosin, original magnification ×100).

The patient's diarrhea resolved within 2 weeks of olmesartan discontinuation. His anemia improved to baseline and he returned to his previous weight within 3 months. Follow-up endoscopy 14 weeks later demonstrated complete resolution of the duodenal inflammatory changes and restoration of normal villous architecture (Figure 2).

## 2. Discussion

Olmesartan is an angiotensin receptor blocker (ARB) commonly prescribed in the management of hypertension. It is associated with few adverse effects, primarily dizziness, although diarrhea is noted in 1–10% of individuals [1]. Severe, sprue-like enteropathy associated with olmesartan was first described by Rubio-Tapia et al. in 2012 [2]. They reported 22 patients on olmesartan for a mean duration of 3.1 years presenting with diarrhea and weight loss. All patients were negative for anti-TTG antibody and were nonresponders to a gluten-free diet. Intestinal biopsies demonstrated villous atrophy in all patients, acute inflammation in 15 patients, and increased intraepithelial lymphocytes in 14 patients. Subepithelial collagen deposition was identified in seven patients. Pathologic changes in other organs included lymphocytic gastritis in five patients, collagenous gastritis in two patients, and microscopic colitis in five patients. Hospitalization for severe dehydration was required for 14 patients. Discontinuation of olmesartan resulted in clinical response in all patients and histopathologic resolution in 17 of 18 patients with follow-up intestinal biopsies performed after a range of 54–707 days. Several other case reports and series have reported similar findings, including a French national case series of 36 patients, with one case of irbesartan-associated enteropathy [3].

In an effort to determine if literature was emphasizing an exceedingly rare reaction to olmesartan or the most severe cases in a clinical spectrum of olmesartan-associated disease, a case-control study of 2,088 patients undergoing esophagogastroduodenoscopy and 12,428 patients undergoing colonoscopy was performed [4]. No association was identified between olmesartan use and diarrhea or histological diagnoses of celiac disease or microscopic colitis, suggesting that olmesartan-associated enteropathy is not part of a broader disease spectrum.

However, a recent retrospective cohort study of patients on ARBs with abdominal pain and no diarrhea reached a different conclusion. Lagana et al. found that 10 of 20 patients with abdominal pain on olmesartan had one or more sprue-like histological features [5]. There was a nonsignificant trend towards more sprue-like histologic features in patients taking olmesartan compared to patients taking other ARBs. This study suggests that there may be a wider spectrum of olmesartan-associated duodenal injury. The mechanism of intestinal injury and whether individuals exhibiting such histopathologic changes are at risk of developing a severe sprue-like enteropathy remains unknown. There are no previous documented cases of olmesartan-associated uveitis.

The case described here demonstrated full symptomatic and pathologic resolution after suspension of olmesartan within a four-month period. Given the life-threatening nature of the enteropathy, no rechallenge was instituted. In the assessment of enteropathy, physicians should be mindful of the broad differential diagnosis including medications, particularly olmesartan. Olmesartan-associated enteropathy may be of particular consideration in patients with seronegative duodenal villous atrophy or celiac disease refractory to gluten exclusion.
